# Impact of age on duration of viral RNA shedding in patients with COVID-19

**DOI:** 10.18632/aging.104114

**Published:** 2020-11-20

**Authors:** Chenliang Zhou, Tianfang Zhang, Haotang Ren, Shanshan Sun, Xia Yu, Jifang Sheng, Yu Shi, Hong Zhao

**Affiliations:** 1 Department of Critical Care Medicine, Renmin Hospital of Wuhan University, Wuhan 430000, China; 2 State Key Laboratory for Diagnosis and Treatment of Infectious Diseases, National Clinical Research Center for Infectious Diseases, Collaborative Innovation Center for Diagnosis and Treatment of Infectious Diseases, The First Affiliated Hospital, College of Medicine, Zhejiang University, Hangzhou 310003, China

**Keywords:** SARS-CoV-2, viral shedding, risk factors, COVID-19

## Abstract

Background: The aim of this study was to investigate the host factors of patients with COVID-19 that were associated with delayed viral RNA clearance in specimens obtained from the upper respiratory tract.

Results: A median of a 32-day period of viral RNA shedding was observed, ranging from 4 days to 111 days. On multivariate analysis, elderly age was independently associated with prolonged viral shedding (OR = 1.02, 95% CI: 1.01–1.04, P = 0.003). An incremental increase in the duration of viral RNA shedding was observed with increasing age (P < 0.05). The median (quartile) duration of viral RNA shedding was 23 (22) days (≤ 40 years), 30 (18) days (41–50 years), 33 (21) days (51–60 years), 34 (17) days (61–70 years) and 34 (17) days (> 70 years).

Conclusions: Viral RNA shedding can persist for as long as 111 days in the upper respiratory tract. Increasing age is associated with viral RNA persistence.

Method: The demographic and virological data of patients with laboratory-confirmed COVID-19 were retrospectively analyzed. A multivariate logistic regression analysis was performed to identify significant risk factors associated with delayed viral RNA clearance. The duration of viral shedding was compared among age-stratified groups.

## INTRODUCTION

Coronavirus disease 2019 (COVID-19) is caused by severe acute respiratory syndrome coronavirus 2 (SARS-CoV-2). This virus poses a global threat that has not been contained. SARS-CoV-2 is the third coronavirus in the past two decades that has crossed species to infect human beings at a high mortality rate [1]. The other two are SARS-CoV-1 [2] and the Middle East respiratory syndrome coronavirus (MERS-CoV) [3]. The dynamics of SARS-CoV-2 viral infection in patients with COVID-19 has been investigated in several recent studies [4–7]. Wölfel et al., reported a detailed virological analysis of nine cases of mild COVID-19 presenting a pharyngeal virus shedding peak during the first week of symptoms, persisting well after the symptoms have ended [4]. This fact is highly relevant in terms of the control of infections in hospitals and discharge management. To further determine the risk factors related to viral RNA shedding**,** Xu et al., observed 113 laboratory-confirmed patients with COVID-19 from two hospitals outside Wuhan and found that prolonged SARS-CoV-2 RNA shedding was associated predominantly in males, of older age, associated with hypertension. Other relevant factors included, delayed admission to the hospital after illness onset, severe illness at admission, invasive mechanical ventilation, and corticosteroid treatment [5]. Moreover, Hu et al., identified a significant relationship between age and duration of RNA shedding through a retrospective cohort study including 59 hospitalized patients with confirmed COVID-19 [6], while Zhou et al., focused on 41 discharged patients with severe COVID-19 and found no difference between patients < 65-years-old and > 65-years-old in duration of RNA shedding [7]. In short, the duration of viral shedding varies greatly among individuals and the associated host factors have not been fully clarified. In the study, potential host factors associated with delayed viral RNA clearance were explored in a retrospective cohort study of 384 patients with COVID-19.

## RESULTS

**Figure 1:**
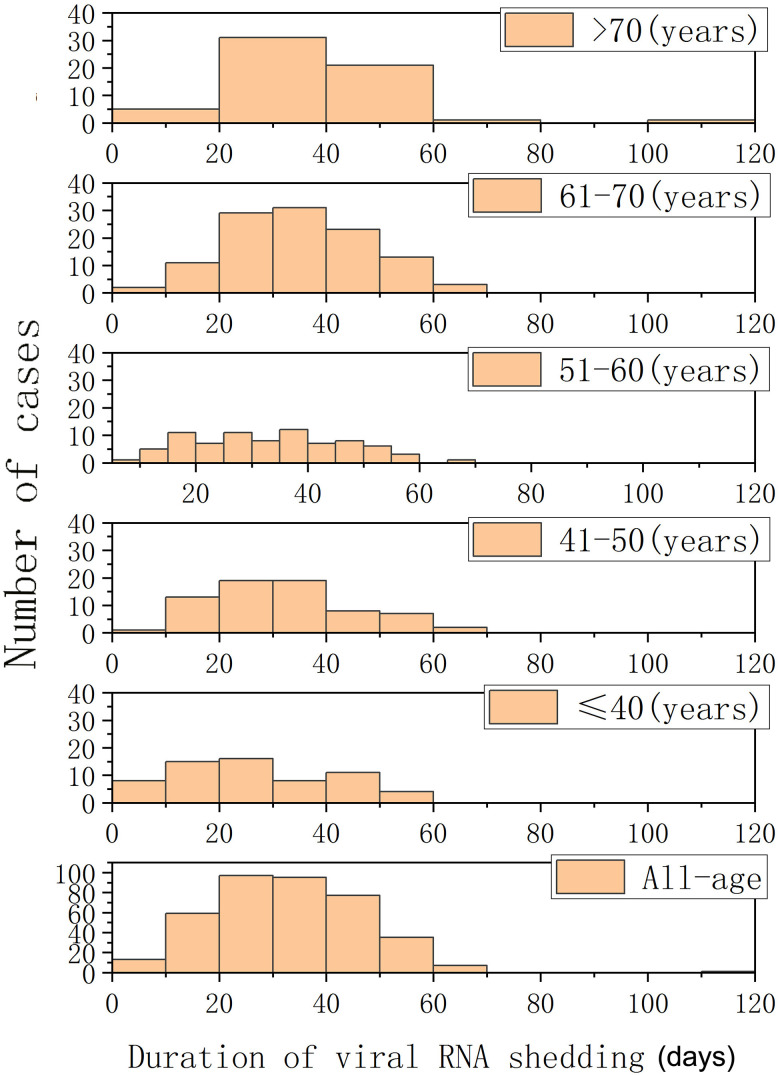
**Histograms of periods of viral RNA shedding (days) in patients with COVID-19 and different age groups.**

As shown in Table 1, 384 COVID-19 patients were included for analysis, with a median age of 58 years. The proportion of males in this study was 46%. Regarding co-morbidities, hypertension was most frequent (32%), followed by diabetes (14.1%) and coronary heart diseases (7.0%). A median 32-day period of viral RNA shedding was observed, ranging from 4 days to 111 days. A multivariate logistic regression was conducted to determine risk factors for viral RNA shedding in COVID-19 patients (data shown in Table 2). No significant statistical difference was shown for sex or co-morbidities (hypertension, diabetes, CHD). Elderly age was the only significant host factor associated with delayed viral RNA clearance (OR = 1.02, 95% CI: 1.01–1.04, P = 0.003). Based on univariate analysis, neither the disease severity nor therapies had any impact on the duration of viral RNA persistence. A subgroup analysis of viral RNA shedding stratified by age is shown in Figure 1. These data revealed an incremental increase in the duration of viral RNA shedding observed with increasing age (P < 0.05).

**Table 1:** Demographic and clinical characteristics of COVID-19 patients.

**Variables**	**Value (N = 384)**
**Demographics**	
Age (years)	59 (23)
Sex (male%)	179 (46.6%)
**Comorbidities**	
Hypertension	123 (32.0%)
Diabetes	54 (14.1%)
Coronary heart disease	27 (7.0%)
**Symptoms**	
Fever	299 (77.9%)
Cough	240 (62.5%)
Expectoration	94 (24.5%)
Pharyngalgia	18 (4.7%)
Fatigue	128 (33.3%)
Dyspena	54 (14.1%)
**Disease severity**	
Mild / Severe / Critical group	157 / 129/ 15
**Antiviral treatment**	
With abidor	258 (67.2%)
With Lianhua Qingwen	199 (51.8%)
With oseltamivir capsules	68 (17.7%)
With hydroxychloroquine, or chloroquine	95 (24.7%)
With ribavirin	135 (35.2%)
With glucocorticoid	113 (29.4%)
**Laboratory detections**	
Leukocyte count (10 ^9^ /L)	5.2 (4.3)
Lymphocyte (10 ^9^ /L)	0.9 (0.3)
Platelet count (10 ^9^ /L)	268 ± 133
Serum bilirubin (μmol/l)	27 ± 20
Creatinine (μmol / L)	55 (28)
**Outcome**	
Duration of detectable viral RNA in sputum (days)	32 ± 21

Data are expressed as the mean ± standard deviation, median (interquartile range) or number (percent). Comparisons between groups were performed using one-way ANOVA, the Mann–Whitney U test, or a chi-squared test.

**Table 2:** Host factors associated with delayed viral RNA clearance.

**Variables**	**Total N(%)**	**Multivariate logistic regression**		
		**OR**	**95%CI**	**P value**
Age	58(22)	1.02	1.01-1.04	0.003
Male	179(46.6)	0.68	0.45-1.03	0.072
Presence of hypertension	123(32.0)	1.21	0.76-1.93	0.41
Presence of diabetes	54(14.1)	0.61	0.33-1.12	0.11
Presence of CHD	27(7.0)	0.96	0.43-2.17	0.93

Statistical analysis was performed using a multivariable logistic regression model. CHD: coronary heart disease.

## DISCUSSION

A retrospectively cohort study was performed to analyze the duration of viral RNA shedding in patients with COVID-19 in order to determine the related risk factors associated with prolonged viral RNA shedding.

This study reported a median of 32 days of viral RNA shedding from illness onset in patients with COVID-19. Most patients showed viral shedding within 60 days. The longest period reached 111 days in a 75-year-old male patient. This is in contrast to a study reporting that viral RNA existed for a median duration of 17 (9) days among 113 patients with COVID-19 [5]. Another study of 59 patients reported that the duration of viral RNA shedding was 26 (7) days. The discrepancies between studies may be attributed to differences in demographic data as well as the severity of the disease. Although it has been demonstrated that viral load in the upper respiratory tract declines after the first week, the persistent viral RNA shedding may impose a risk of transmission over the long-term. Therefore, discharged patients still require close monitoring of viral load to effectively block the transmission of the virus. Further studies are needed to ascertain whether live virus particles are shed even in the later stages of COVID-19.

A multivariate logistic regression analysis showed that among host factors, no statistically significant relationship was found among sex or co-morbidities (hypertension, diabetes, CHD) on the duration of viral RNA shedding. Elderly age was the only host factor that was significantly associated with prolonged viral RNA shedding in the present study. The effect of age on viral RNA clearance might be conflated by other factors because elderly patients are more likely to have co-morbidities and more severe pre-existing diseases [8, 9]. However, in our study, disease severity had no impact on the duration of viral RNA persistence based on the univariate analysis. The impact of age remained significant by adjusting co-morbidities. The effect of different regimens and viral RNA persistence was found not to be statistically different between groups stratified by any antiviral therapies. The delayed viral RNA clearance in elderly patients is likely linked to the detrimental effect of aging on lymphocyte development and function [10]. Elderly patients sustain atrophy of the thymus combined with the normal decrease in the number of T lymphocytes with aging. In addition, the activation potential of memory T cells that accumulate is altered, leading to hyporesponsivity in host immune responses. Thus, normal age-related impaired immunity was presented in elderly patients with COVID-19 [11]. In that case, an age-related decline of innate and adaptive immunity leads to a reduced ability to respond to infection, which might result in a diminished clearance of RNA virus in elderly patients. Conversely, elderly patients with COVID-19 usually face a greater risk of morbidity, in addition to more frequent co-morbidities in elderly patients related to the prognosis of COVID-19. The findings presented here on impaired viral RNA clearance may also provide a reasonable explanation for the higher incidence of rapid disease progression and death in elderly patients. The age-related impact of viral RNA clearance may also explain the discrepancy in our findings and other studies as previous described, showing a median age of 58 years in the present study compared with studies reporting a median of 17 days in 113 patients of a median age of 52 years [5] and 26 days in 59 patients of a median age of 46 years, respectively [6].

In conclusion, we found that viral RNA shedding in upper respiratory tract specimens can persist for as long as 111 days. Elderly patients were associated with delayed viral RNA clearance. Therefore, more attention should be given to discharged patients, especially elderly patients, to conduct nucleic acid detection in order to better control the progression of the disease and to prevent recurrence and transmission of COVID-19.

## MATERIALS AND METHODS

Demographic data (age, sex, and underlying comorbidities) and virological data (duration of viral RNA clearance) were retrospectively collected from patients with laboratory-confirmed COVID-19 who were admitted from January 11, 2020 to March 24, 2020 at Renmin Hospital, Wuhan University, and were placed in a subgroup for analysis by varying age. SARS-CoV-2 RNA in nasopharyngeal or oropharyngeal swab specimens was detected by a real-time RT-PCR approach as previously described [2] every 2 to 4 days. The duration of RNA viral shedding was measured from the onset of symptoms to three consecutive negative monitorings. Demographic data were collected through electronic medical records. All hospitalized patients received a standard treatment based on the New Coronavirus Pneumonia Prevention and Control Program (7^th^ edition) [12]. The study complied with the principles of the Declaration of Helsinki and was approved by the Ethics Committee of Renmin Hospital of Wuhan University and First Affiliated Hospital of Zhejiang University. Consent was obtained from all patients or their authorized representatives.

### Statistical analysis

Continuous variables are represented as the median (interquartile range) and nominal variables are expressed as a number (percentage). A comparison of the viral RNA shedding period among different groups was performed with the Mann-Whitney U test. Host factors associated with prolonged viral RNA shedding were explored by a multivariate logistic regression and were considered significant if a two-sided P-value was P < 0.05. Statistical analysis was performed with SPSS 20.0 (SPSS Inc.; Chicago, IL, USA).

### Ethics approval and consent to participate

This study was reviewed and approved by the Ethics Committee of Renmin Hospital, Wuhan University and the First Affiliated Hospital of Zhejiang University. Following a full explanation of the study, written consent was obtained from all patients or their authorized representatives.

### Availability of data and materials

The datasets and materials used for the study are available from the corresponding author upon reasonable request.
